# Identification of novel prognostic indicators for triple-negative breast cancer patients through integrative analysis of cancer genomics data and protein interactome data

**DOI:** 10.18632/oncotarget.12287

**Published:** 2016-09-27

**Authors:** Fan Zhang, Chunyan Ren, Hengqiang Zhao, Lei Yang, Fei Su, Ming-Ming Zhou, Junwei Han, Eric A. Sobie, Martin J. Walsh

**Affiliations:** ^1^ College of Bioinformatics Science and Technology, Harbin Medical University, Harbin 150086, PR China; ^2^ Department of Pharmacological Sciences, Icahn School of Medicine at Mount Sinai, New York, NY 10029, USA

**Keywords:** triple-negative breast cancer, landmark for cancer prognosis, GTPase, ubiquitination, oxidative damage

## Abstract

Triple negative breast cancers (TNBCs) are highly heterogeneous and aggressive without targeted treatment. Here, we aim to systematically dissect TNBCs from a prognosis point of view by building a subnetwork atlas for TNBC prognosis through integrating multi-dimensional cancer genomics data from The Cancer Genome Atlas (TCGA) project and the interactome data from three different interaction networks. The subnetworks are represented as the protein-protein interaction modules perturbed by multiple genetic and epigenetic interacting mechanisms contributing to patient survival. Predictive power of these subnetwork-derived prognostic models is evaluated using Monte Carlo cross-validation and the concordance index (C-index). We uncover subnetwork biomarkers of low oncogenic GTPase activity, low ubiquitin/proteasome degradation, effective protection from oxidative damage, and tightly immune response are linked to better prognosis. Such a systematic approach to integrate massive amount of cancer genomics data into clinical practice for TNBC prognosis can effectively dissect the molecular mechanisms underlying TNBC patient outcomes and provide potential opportunities to optimize treatment and develop therapeutics.

## INTRODUCTION

TNBCs refer to certain breast cancers negative of estrogen receptor (EsR) and progesterone receptor (PgR) expression, as well as Her-2/Neu receptor overexpression [1]. They are significantly associated with younger age, African American and Hispanic ethnicities, more aggressiveness, higher distant recurrence, and poorer survival than other breast cancers [2, 3]. Traditional hormone-based therapy (i.e. tamoxifen to target EsR positive cells), and antibody-based therapy (i.e. trastuzumab to target Her2/Neu positive breast cancers) are not effective when treating TNBCs [4]. In the past several years, multiple clinical trials have focused on TNBCs [5–7], but all failed, despite a few ongoing clinical trials show some promising results [1]. Meanwhile, multiple studies have focused on finding prognostic and/or therapeutic markers. Some common markers have been identified, such as basal cytokeratin (CK) 5/6 and epidermal growth factor receptor (EGFR) [8, 9]. Recently, microRNAs and lncRNAs have become emerging targets to predict cancer prognosis and classify patients for clinical treatment [10–13]. However, chemotherapy still remains as the only clinical option for TNBCs [4].

It is well known that cancer is a complex disease because of combined effects of multiple genetic and epigenetic changes and subsequent dys-regulation of critical signaling pathways [14]. The heterogeneity of TNBCs further exacerbates the problem to identify prognostic and/or therapeutic markers [1]. Pioneering molecular portraits of human breast cancer have provided us invaluable information about the link between gene expression pattern and phenotypic diversity [15]. Similarly, pioneering prognosis work for breast cancer patients mainly used gene expression signatures [16, 17] combined with various analytic approaches such as meta-analysis, functional enrichment analysis, and transcriptional network analysis [18]. Thanks to the recent advances in next-generation sequencing technology, we are able to acquire multi-dimensional genomic data with clinical information from a large number of patient samples from Cancer Genome Atlas (TCGA) including breast cancer [19], and dissect cancers beyond the traditional clinical variables (i.e. age and tumor stage) by incorporating multi-layered data to represent genomic activity at different levels, such as gene expression profiles, gene copy number variants (CNVs), miRNA expression, DNA methylation states and molecular interaction networks. Such integrated analyses using multi-layered molecular information have been performed to help understand cancer outcomes [20, 21].

Here, we seek to identify prognostic markers using a network-based approach through integrative analysis of TCGA cancer genomics data due to the hypothesis that multiple genetic and epigenetic events together lead to a complex TNBC outcome. In this work, we proposed a systematic methodology to predict cancer prognosis: (1) Score each gene based on the synthetic effect of different molecular features (mRNA expression, CNV, and DNA methylation) on patient overall survival, (2) Identify novel subnetwork signatures correlated with patient survival, (3) Assess the prognostic power of the subnetwork signatures, and (4) Evaluate the utility of these subnetwork-derived models in TNBC prognosis through functional enrichment analysis, tumor stratification, and independent validation. We find subnetworks related to low oncogenic GTPase activity, low ubiquitin/proteasome degradation, effective protection from oxidative damage, low PIK3A activity, and tightly immune response are linked to better prognosis. Several biosynthesis and metabolism related subnetworks have also been identified. We expect that such a systems biology/precision medicine approach to integrate cancer genomics and interactome data can be useful to understand mechanisms underlying TNBC prognosis and benefit clinical TNBC management.

## RESULTS

### Discovery of network-based prognostic biomarkers

We extracted the molecular features (including mRNA, CNV, and DNA methylation), clinical variables (i.e. age, tumor stage, and grade), and overall survival information of 119 TNBC patients and 583 non-TNBC patients in TCGA. TNBC and non-TNBC patients were separated according to the EsR, PsR, and HER2 status (Table 1). Mutation was not included because it happens rarely across the whole genome despite some common mutations (i.e. TP53), which can be considered as a clinical variable and left as an indicator for predication evaluation or subtyping. miRNA was not included neither because of the insufficient samples left for analysis.

**Table 1:** Summary of the three primary molecular features derived from specimens of BRCA in TCGA by high throughput analysis

Cancer	CNV	Methylation	mRNA	Core set
Non-TNBC	gistic2	450k/27k (combined)	HiseqV2	
	583 × 11,878 genes	595 × 19847 probes	595 × 11,442 genes	583 × 43167
TNBC	gistic2	450k/27k (combined)	HiseqV2	
	119 × 11,878 genes	123 × 19847 probes	123 × 11,442 genes	119 × 43284

**Figure 1:**
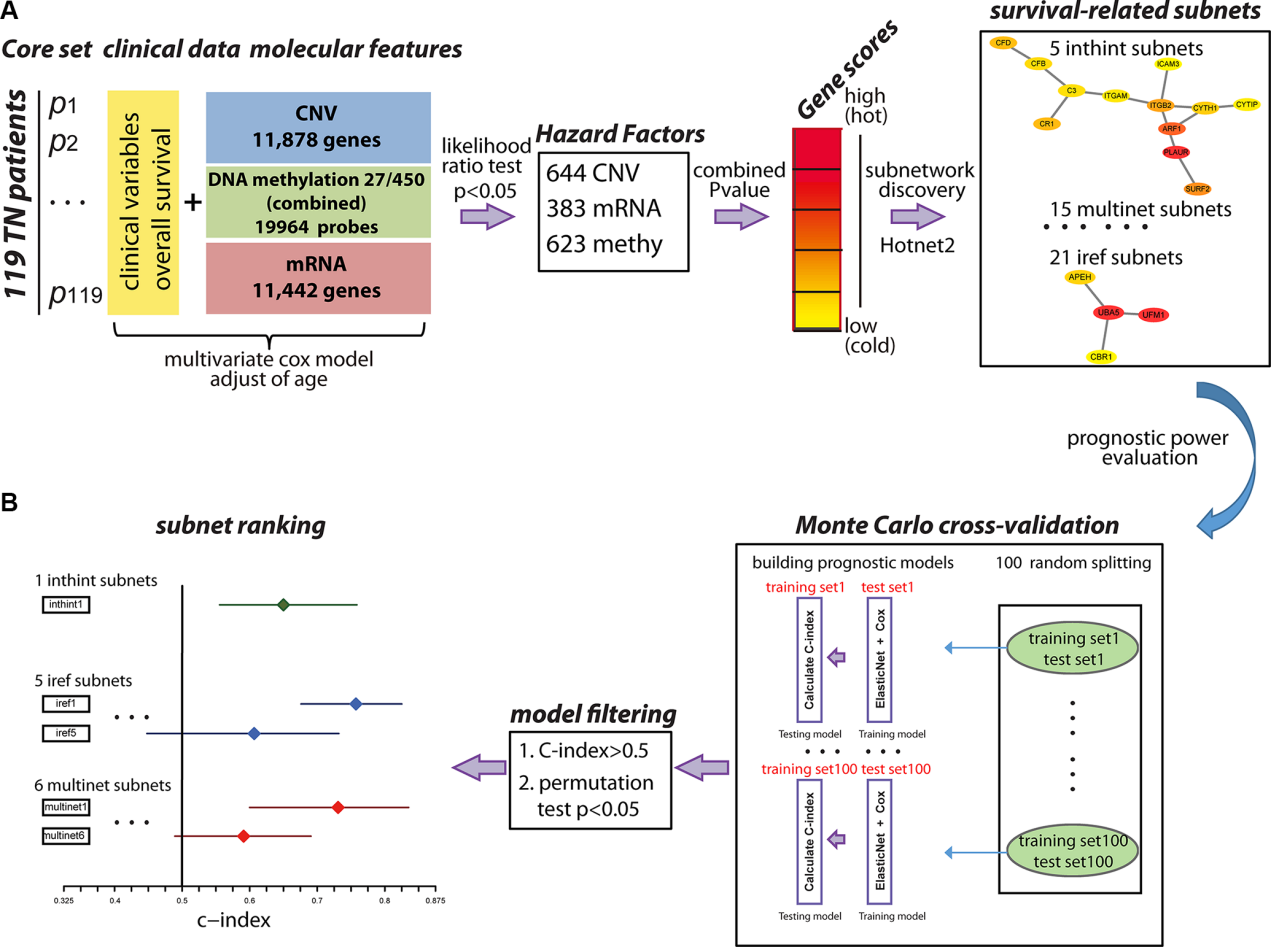
An overview of the methodology (**A**). Identification of survival-related subnetwork biomarkers. First, molecular features (CNV, mRNA, DNA methylation) and clinical variables from 119 TNBC patients were collected as the core set. Next, multivariate cox model was used to select hazard factors (644 CNV, 383 mRNA, 623 DNA methylation) filtered by likelihood ratio test *p*-value < 0.05, which represented the significance of each molecular feature correlated with patient overall survival adjusted for age. Furthermore, the heat score for each gene was calculated as the negative sum of the natural logarithm of the single molecular feature *p*-values (Red: high score; Yellow: low score) to evaluate the collaborative effect of different molecular features on patient overall survival. Subnetworks were identified using HotNet2 algorithm in three PPI networks using a heat diffusion process and a statistical test based on both the score of the genes and the local topology of the subnetwork. (**B**). Evaluation of the multi-dimensional subnetwork-derived prognostic models. Monte Carlo cross-validation and C-index were applied to assess the predictive power of each subnetwork signature. During each of the 100 times of random splitting, 80% of the total samples were used to train the model and the remaining of 20% were used as the test set for C-index calculation. C-index > 0.5 and permutation test *p* < 0.05 were applied as the filtering criteria. C-index for each subnetwork was plotted with the median in the center and the whiskers marking the 25% and 75% percentile. The vertical black line marked the C-index equivalent to a random guess (C-index = 0.5).

**Figure 2:**
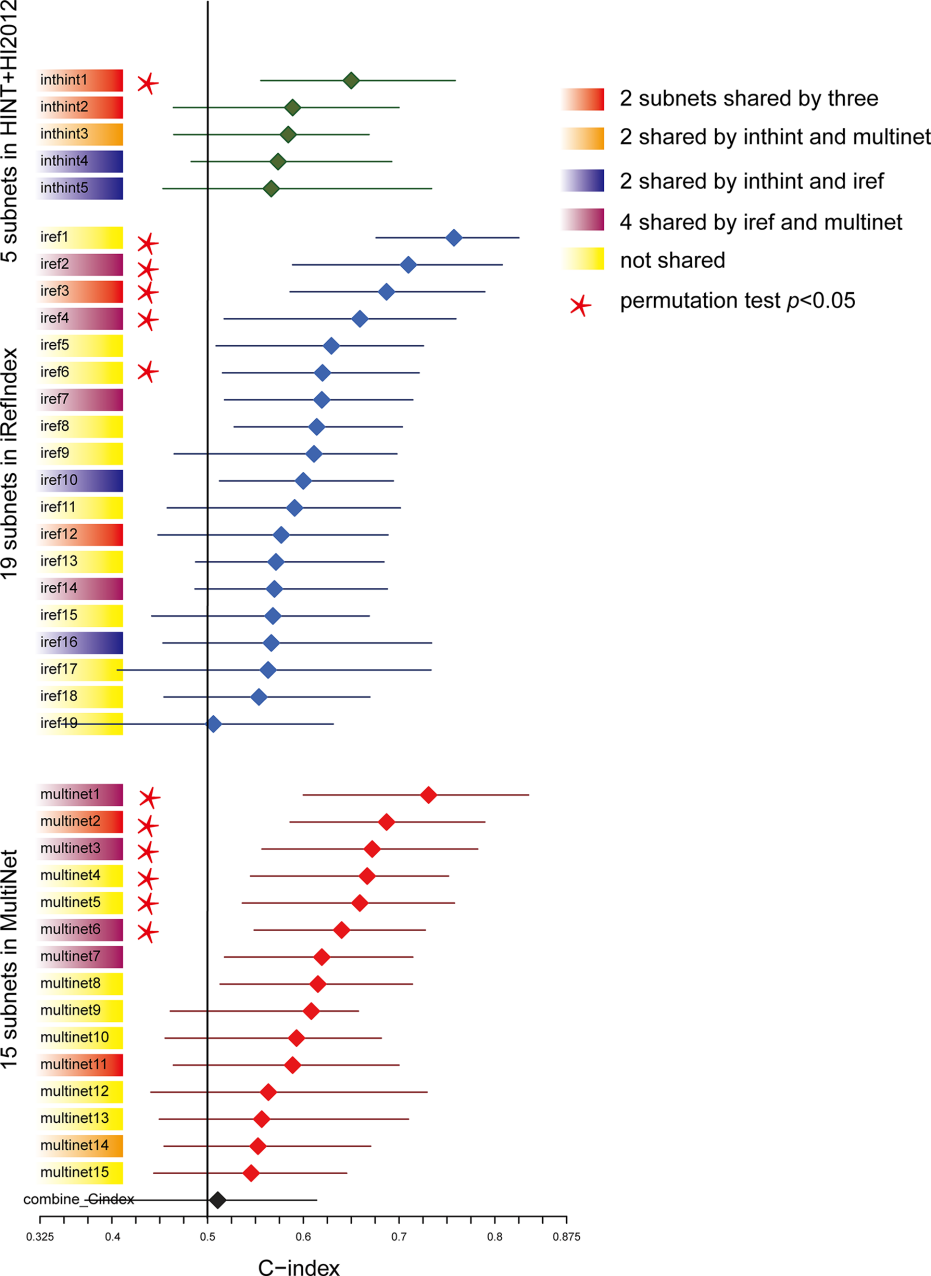
Predictive power ranking of the survival related subnetwork biomarkers Subnetworks were numbered from 1 to N according to the predictive power in descending order. C-index for each subnetwork was plotted with the median in the center and the whiskers marking the 25% and 75% percentile. The vertical black line marked the C-index equivalent to a random guess (C-index = 0.5). Subnetworks shared by different PPIs were colored (Red: share by three; Orange: shared by HINT+HI2012and MultiNet; Dark purple: share by HINT+HI2012and iRefIndex; Plum: shared by iRefIndex and MultiNet; Yellow: not shared). Subnetworks passing the permutation test (*p* < 0.05) based on the comparison of the median C-index values of the original survival data with the distributions of the median C-indexes of the 100 permuted survival data were labeled with a star.

**Figure 3:**
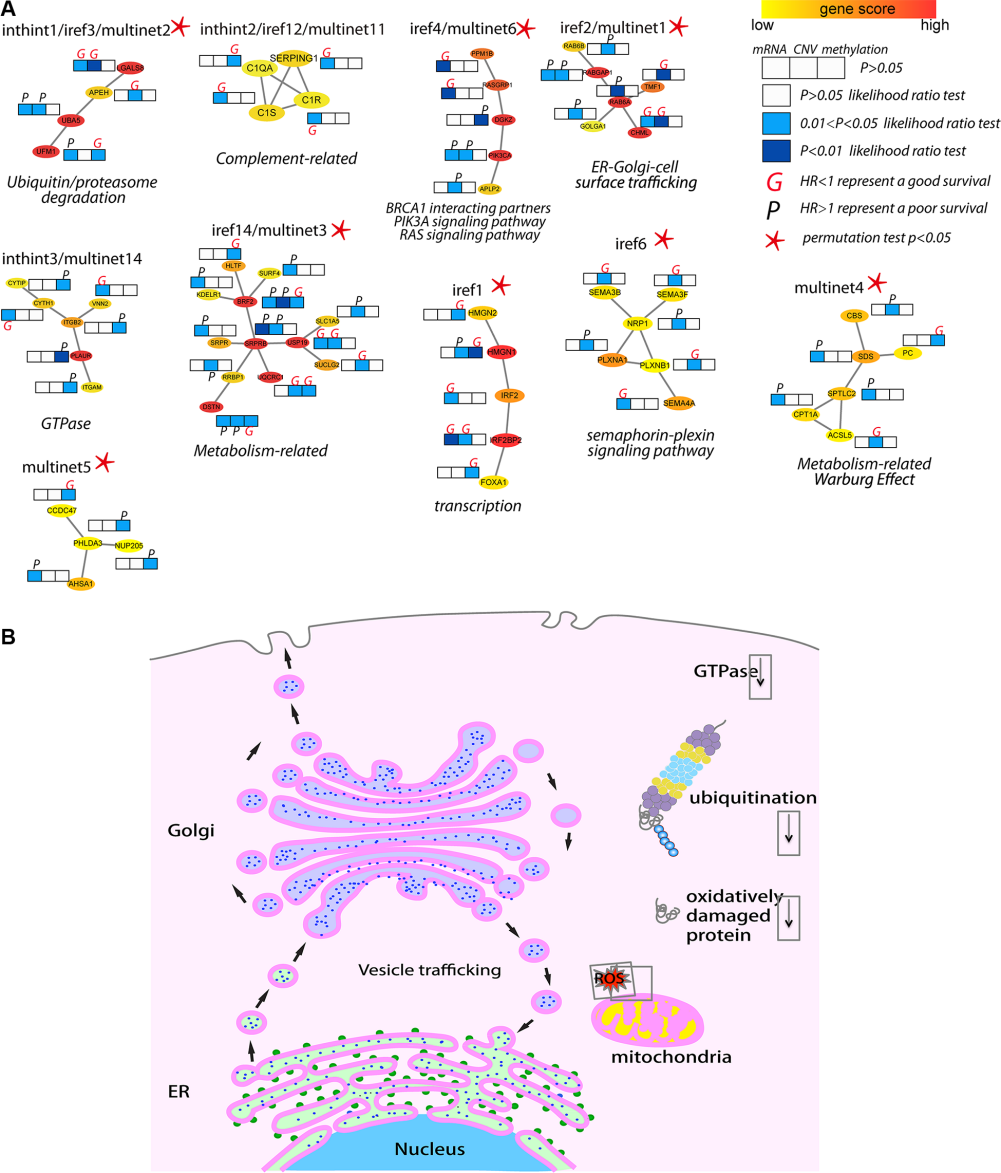
Biological insights of the top/shared prognostic subnetwork biomarkers (**A**) Top ranked and shared subnetwork biomarkers as examples to illustrate the molecular insights from the prognostic models. Subnetworks were plotted showing the interactions among genes with the color representing the heat score (Red, hot: higher significance; Yellow, cold: less low significance). Multi-dimensional information (mRNA, CNV and DNA methylation) of each gene in each subnetwork associated with survival outcome (derived from the multivariate Cox regression analysis in 119 TNBC patients) was plotted as likelihood ratio test *p*-value represented as color (white, light blue and dark blue). HR was used to estimate the association of individual molecular feature with survival (better or worse), where an HR greater (or less) than 1 represented a worse (or better) prognosis and marked as “P” (or “G”). (**B**) A schematic view of the major signaling pathways and cell organelles significantly related to TNBC survival. Functioning mitochondrion, ER, Golgi, and vesicle trafficking among ER, Golgi and cell surface, as well as low ubiquitin/proteasome activity and oxidative damaged proteins are linked to better survival. Cell organelles: Nucleus (cyan), ER (light green), ribosomes (green), Golgi apparatus (light purple), mitochondrion (yellow). Cellular activities: ubiquitin (maroon), proteasome (grey, yellow, cyan), ROS (red), oxidative damaged protein (red curls).

Considering the fact that multiple genes and multiple levels of regulation may function together to generate the complex cancer outcome, we adopted a network-based approach to generate multi-dimensional subnetwork-based predictors for the prognosis of TNBC (Figure 1). First, we did a pre-selection step using a multivariate Cox proportional hazards model to estimate the effect of different molecular features on patient overall survival time. In total, we selected 1,650 features as hazard factors, including 383 mRNA expression changes, 623 promoter DNA methylation changes, and CNV of 644 genes, which mapped to total 1,487 survival-related genes (*p* < 0.05 as the significance cutoff from the likelihood ratio test). Next, we derived a score (heat) for each of the genes through the Equation (1) as the input into HotNet2 [22, 23], which uses a heat diffusion process and a statistical test based algorithm to discover subnetwork signatures in three PPI networks (HINT [24]+ HI-2012 [25], iRefIndex [26], and MultiNet [27]). Totally, 41 altered subnetworks with > = 4 nodes were detected. Furthermore, we assessed the predictive power of these subnetwork-based molecular signatures using the median C-index calculated from the Monte Carlo cross-validation (MATERIALS AND METHODS). The nonparametric C-index is scaled such that a C-index of 1 indicates perfect prediction accuracy, whereas a C-index of 0.5 is equal to random guess. We identified 39 out of the 41 subnetworks with a C-index > 0.5 and multiple subnetworks were detected in more than one PPI network (i.e. inthint1/iref3/multinet2, iref2/multinet1, iref4/multinet6) (Figure 2). These subnetworks were numbered from 1 to N according to the predictive power in descending order (Figure 2 and Supplementary Tables S1–S3). To further understand how these subnetwork biomarkers may be related to patient survival, we investigated individual gene using NCBI Entrez Gene [28] and literature search and performed functional enrichment analysis based on the known pathways or ontologies using Enrichr [29]. Such a thorough analysis identified several pathways known to be involved in TNBC such as iref4/multinet6, which contain BRCA1 interacting partners and PIK3A signaling pathway [30]. Several metabolism-related subnetworks have also been identified, such as multinet3 and multinet4/8/9/12. Most importantly, we found quite large amount of a subnetworks related to GTPase, endoplasmic reticulum (ER)-Golgi-cell surface trafficking, ubiquitin/proteasome system, and complement system (Figure 3 and S1 text).

Furthermore, we calculated *P*-values based on the comparison of the median C-index values of the original survival data with the distributions of the median C-indexes of the 100 permuted survival data to test if the models were statistically significant (C-index > 0.5, survival-data permutation test *p* < 0.05 as filter criteria). Finally, we determined 1 subnetwork in HINT+HI2012 (inthint1), 5 in iRefIndex (iref1 ~ iref4, iref6), 6 in MultiNet (multinet1 ~ multinet6) as candidate prognostic biomarkers significantly associated with patient overall survival, which were labeled with a star in Figure 2 and plotted in detail in Figure 3A.

### Independent validation

**Figure 4:**
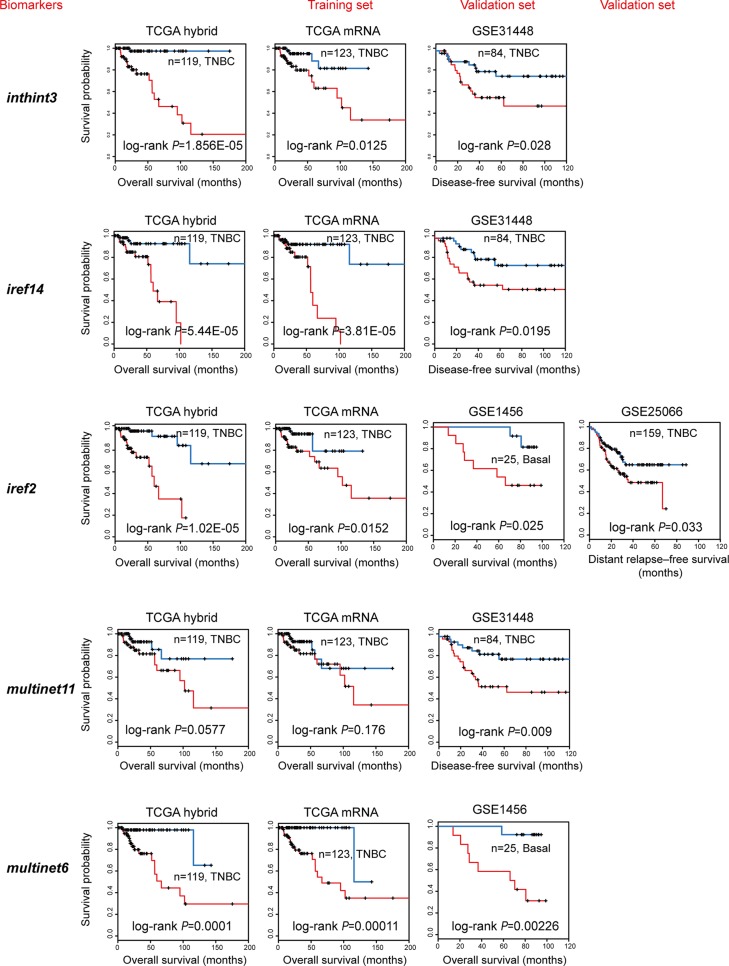
Independent validation of the subnetwork biomarkers obtained from TCGA Multivariate Cox regression analysis of overall survival was performed using TCGA mRNA data (training set, TCGA mRNA) of genes in subnetwork biomarkers to get the estimated regression coefficients to build up a prognostic model. These models were further validated using the TNBC samples from four GEO data sets (validation set). Kaplan-Meier curves showed effective separation of patient survival for GEO data sets using exemplary subnetwork-derived biomarkers from TCGA. TCGA hybrid: patient overall survival analysis using combined mRNA, CNV, methylation data (for features); TCGA mRNA (training set): patient overall survival analysis to generate prognostic models using TCGA mRNA data alone; GEO data sets (test sets, including GSE1456, GSE31448, GSE16446, and GSE25066): patient overall, disease-free, or distant relapse-free survival to validate prognostic models trained by TCGA mRNA data.

Given the limited availability of suitable independent data providing the highly integrated and multi-dimensional genomic data, we validated our subnetwork biomarkers using the most common and accessible gene expression data only. For each subnetwork biomarker, as previously described [31], a risk score model was developed as a linear combination of the mRNA expression levels of the genes in the subnetwork and the estimated regression coefficients in the multivariate Cox regression analysis as the weight (MATERIALS AND METHODS). We were able to calculate a subnetwork-based risk score (referred to as “snRS”) for each patient in the 119 TCGA TNBC samples and classified them into high-risk or low-risk group using the median risk score as the cutoff, The same score model obtained from the discovery series was used to calculate the snRS for each patient from four independent data sets (GSE1456, GSE31448, GSE16446 and GSE25066) (Table 2). We found that the snRSs for 10 subnetwork biomarkers were significantly associated with survival in at least one data set tested and most of the top/shared subnetworks described above were validated (Figure 4). Particularly, iref2/multinet1, the subnetwork containing RAB protein and its inhibitor CHML was significant in TCGA (log-rank *p* = 0.0152), GSE1456 (log-rank *p* = 0.025), and GSE25066 (log-rank *p* = 0.033). Such analysis validated that our subnetwork biomarkers were well predictive of TNBC survival.

**Table 2:** Four breast cancer data sets used for independent validation

Data set	TNBC definition	Sample size	Cancer type	Microarray platform	Reference
GSE25066	EsR-, Her2-, PgR-	159	stage I–III breast cancers	Affymetrix Hu133A	[54]
GSE16446	EsR-, Her2-	80	Primary breast cancer	Affymetrix HU133 Plus 2.0	[55]
GSE31448	EsR-, Her2-, PgR-	84	invasive adenocarcinoma	Affymetrix HU133 Plus 2.0	[56]
GSE1456	Basal-like	25	Primary breast cancer	Affymetrix Hu133A	[57]

### Prognostic *value* of the subnetwork-based biomarkers to assess TNBC clinical outcome

To assess whether the prognostic values of the subnetwork signatures are independent of conventional clinical factors of TNBC patients, we performed the univariate and multivariate analysis using the snRS of iref2 (or multinet6) or other clinical factors as explanatory variables. The result indicated that the snRS maintained an independent correlation with overall survival after adjusting for conventional clinical factors, including age, stage, number of lymph nodes, tumor weight, as shown in Table 3. For example, iref2, as an independent risk factor, was significantly associated with overall survival of TNBC patients (HR = 4.2384, 95% CI: 2.19255–8.193, *p* = 1.75E-05). Taken together, these analyses demonstrated the added value of the subnetwork-based biomarkers in a prognostic setting.

**Table 3:** Univariate and multivariate Cox regression analysis of overall survival in TCGA TNBC dataset

Variables	Univariable model			Multivariable model		
	HR	95% CI of HR	*p* value	HR	95% CI of HR	*p* value
iref2 biomarker						
snRS	2.718	1.878–3.934	1.15E-07	4.2384	2.19255–8.193	1.75E-05
age	1.008	0.9734–1.044	0.653	1.0556	0.99871–1.116	0.0556
stage(III/IV vs.I/II)	4.439	1.728–11.4	0.001962	54.1785	3.68274–797.045	0.00361
Lymph node count	1.808	0.6515–5.018	0.255	0.2453	0.04945–1.217	0.0854
tumor_weight	0.9993	0.9964–1.002	0.627	1.0025	0.99823–1.007	0.253
**multinet6 biomarker**						
snRS	2.72	1.715–4.308	2.09E-05	2.36	1.4122–3.943	1.05E-03
age	1.008	0.9734–1.044	0.653	1.001	0.9604–1.044	0.945
stage(III/IV vs.I/II)	4.439	1.728–11.4	0.001962	9.229	1.6767–50.801	0.0107
Lymph node count	1.808	0.6515–5.018	0.255	1.059	0.2904–3.865	0.93
tumor_weight	0.9993	0.9964–1.002	0.627	1.001	0.9976–1.004	0.614

Abbreviations: HR, hazard ratio; CI, confidence interval

### Comparison with molecular feature-based prognostic predictor

**Figure 5:**
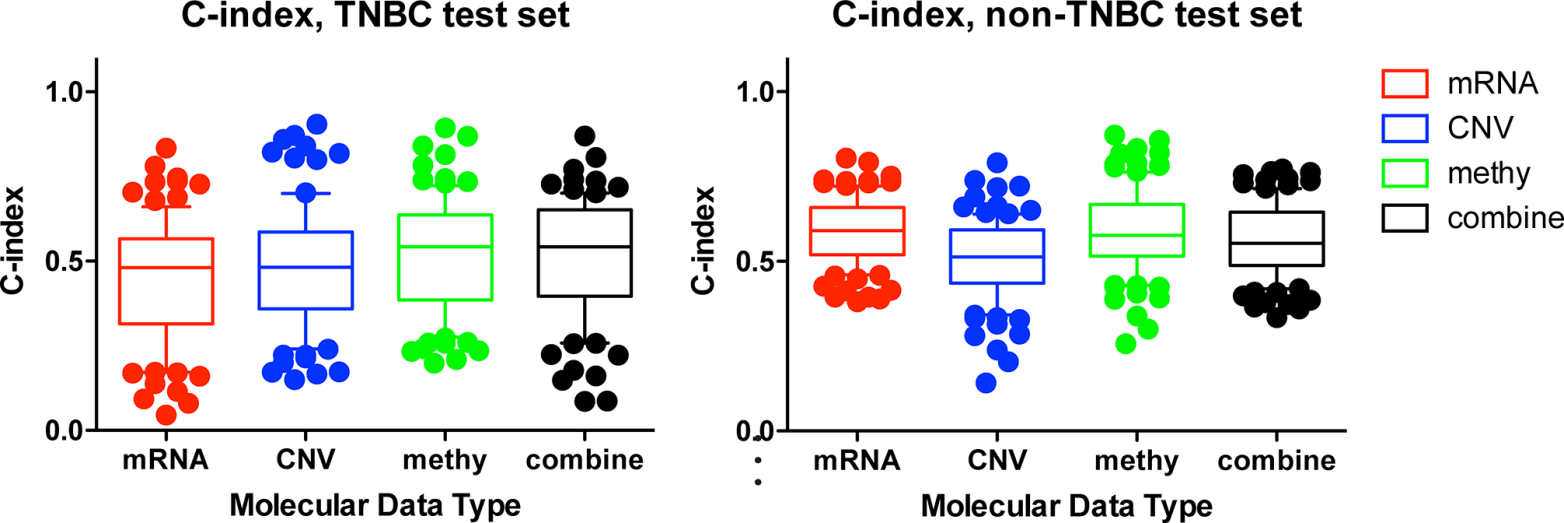
Comparison of the prognostic power among individual and combined molecular data types C-indexes of models trained from individual molecular data alone or combination of multiple data types in TNBC (a), and non-TNBC (b) distinguished with different colors. For each data type, during each of the 100 times of random splitting, 80% of the total samples were used to train the model and the remaining 20% as the test set for C-index calculations. The boxplot was plotted with the median C-index in the center and the whiskers marking the 25% and 75% percentile. The mRNA had the highest C-index in non-TNBC, while the combined molecular data had the highest C-index for TNBC. To compare the performance across different prognostic models, one tailed Wilcoxon signed rank test was used to calculate the *p*-value.

To test our hypothesis that incorporating prior knowledge of cellular protein-protein interactions could enhance the biological insight and predict utility of TCGA genomic data, we compared our network-based method with the molecular feature-based method. To assess the predictive power of individual molecular data type or the combined profiling of multiple types of molecular data, we used the same procedure as described in Yuan's study (which could be accessed in Synapse (doi:10.7303/syn1710282) [20] (MATERIALS AND METHODS). We observed that mRNA data only could hardly predict TNBC patient survival (C-Index = 0.44), while DNA methylation data and combined molecular data (mRNA + CNV + DNA methylation) have improved prognostic power (C-Index = 0.52 and 0.51 respectively). In contrast, mRNA is the best data type to predict the survival of non-TNBC (C-Index = 0.59) (Figure 5). Such analysis indicated that each molecular data type contributed to TNBC prognosis and combining all types of data could lead to better but limited prognostic predictive power. However, when using our subnetwork-based biomarkers, the predictive power was significantly improved (median C-index = 0.7573 for iref1 vs median C-index = 0.51 for combined molecular-based model, one-sided Wilcoxon signed rank test, *p* < 0.0019). Therefore, our subnetwork biomarkers based on biologically related genes and interactions are more meaningful than molecular feature based biomarkers merely selected by LASSO throughout the genome.

### Application of the network-based biomarkers for survival oriented TNBC stratification

**Figure 6:**
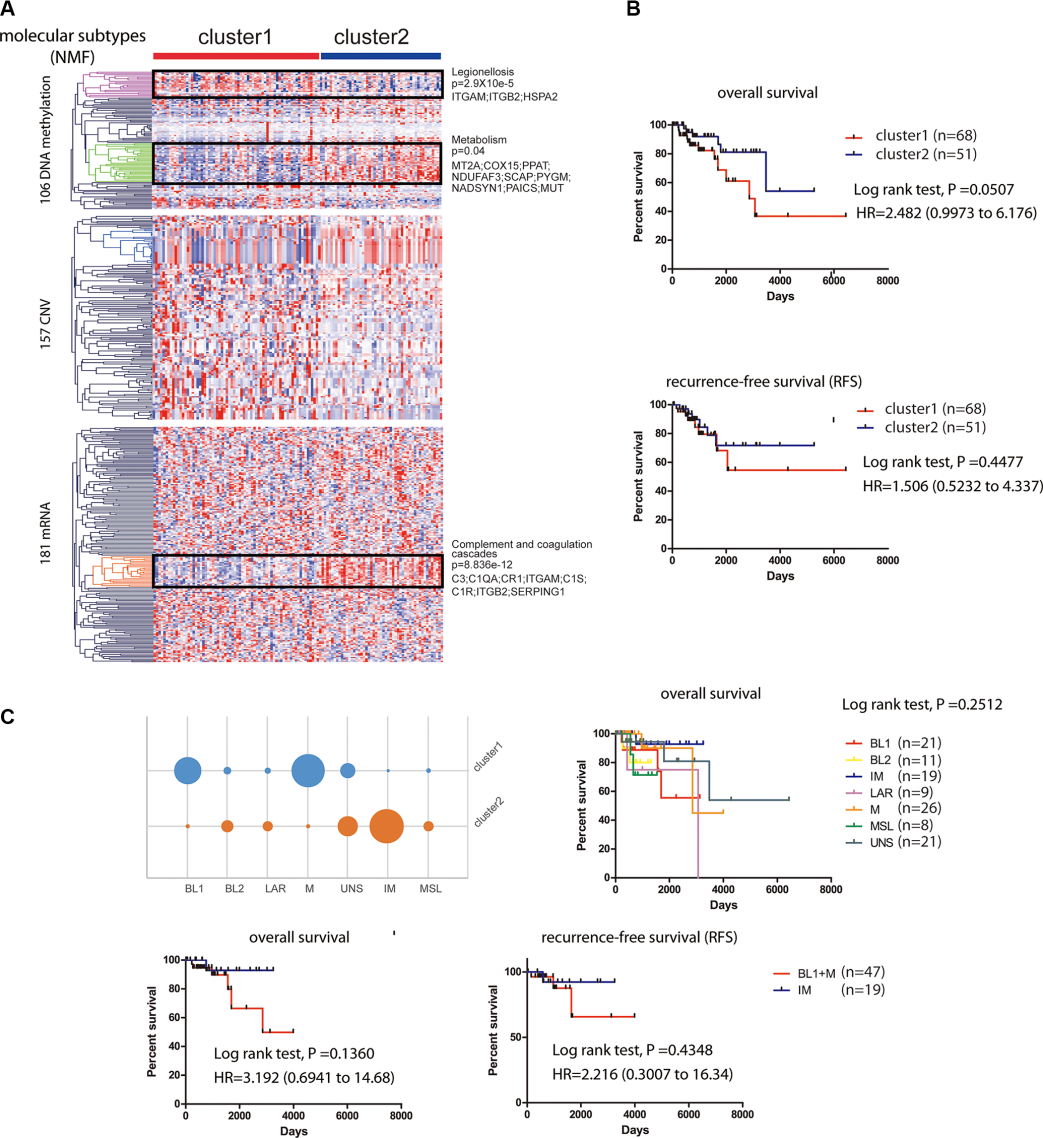
Survival related tumor stratification (**A**). Consensus non-negative matrix factorization (NMF) clustering of 119 KIRC patients based on 444 molecular features (CNV: 157; mRNA: 181; DNA methylation: 106) derived from 39 subnetwork-based prognostic models revealed two molecular subtypes (clusters). Three most different molecular clusters between the high-risk and low-risk groups were highlighted: a DNA hyper-methylation cluster (Legionellosis, poor survival, *p* = 2.9×10e-5), a DNA hypo-methylation cluster (Metabolism, *p* = 0.04), and an mRNA cluster (complement and coagulation cascades, good survival, *p* = 8.836e-12). (**B**). Kaplan-Meier curves showing patients in cluster 2 with better overall survival and recurrence-free survival (RFS) than patients in cluster 1. (**C**). Subtyping 116 TCGA TNBC patients based on Lehmann's method and survival analysis. TNBC patients were subtyped using web-based tool (http://cbc.mc.vanderbilt.edu/tnbc/). Kaplan-Meier curves of overall survival were plotted to show the prognostic difference for six Lehmann subtypes (log-rank test, *p* = 0.2512) (upper panel). IM subtype was stratified to cluster 2 while BL1 and M subtypes were stratified to cluster 1, with better or worse prognosis respectively shown by Kaplan-Meier curves of overall survival and RFS (lower panel).

We further stratified the 119 TNBC patients into high-risk and low-risk groups (HR = 2.482; 95% CI, 0.9973 to 6.176; *p* = 0.0507) via non-negative matrix factorization (NMF) based on the similarity of their molecular profiles, which included 444 molecular features (CNV: 157, methylation: 106, mRNA: 181) derived from the 39 subnetwork models with a C-index > 0.5 (MATERIALS AND METHODS). We found that cluster 2 has better prognosis than cluster 1 on overall survival and recurrence-free survival (RFS) (Figure 6A and 6B). Based on the hierarchical clustering results, we found three most different molecular clusters between the high-risk and low-risk groups: a DNA methylation cluster with hyper-methylation in poor survival group enriched with genes in Legionellosis (*p* = 2.9×10e-5) and a hypo-methylation cluster enriched of genes in Metabolism (*p* = 0.04), as well as an mRNA cluster with higher expression level of the genes enriched in complement and coagulation cascades (*p* = 8.836e-12) in good survival group. Furthermore, we categorized our 116 TNBC samples to the six subtypes using a web-based subtyping tool (http://cbc.mc.vanderbilt.edu/tnbc/) based on a comprehensive study performed by Lehmann et al., which categorized TNBC to six subtypes from 587 TNBC cases based on gene expression profile [32]. Three samples were filtered out based on their sample checking criteria. Interestingly, though Lehmann subtypes were not determined by prognosis (log-rank *p* = 0.2512), our method categorized that IM subtype mainly belonged to cluster 2 while BL1 and M subtypes belong to cluster 1, with better or worse prognosis respectively (Figure 6C).

## DISCUSSION

In this study, we developed a network based method combining multidimensional genetic and epigenetic information as well as clinical data to dissect the mechanisms underlying overall survival of TNBC patients. We identified subnetwork signatures containing important genes, interacting partners, and regulating patterns linked to patient overall survival. We found that low oncogenic GTPase activity, low ubiquitin/proteasome degradation, effective protection from oxidative damage, and tightly immune response were linked to better prognosis. We also confirmed low PIK3A activity and revealed certain metabolism features related to better prognosis. To achieve a prognosis-favor environment, ER and Golgi are of particular importance (Figure 3B).

Here, we particularly would like to discuss the biological insights from two subnetworks while more literature based and functional enrichment-based analyses can be found in supporting information (S1 text).

### inthint1/iref3/multinet2

UBA5 (ubiquitin like modifier activating enzyme 5) and UFM1 (ubiquitin-fold modifier 1) were identified as a novel protein-conjugating system, knockdown or depletion of which could lead to unfolded protein response (UPR), ER stress, and inhibited vesicle trafficking [33, 34]. Knockdown of UBA5 has been shown to inhibit breast cancer cell growth [35], implying the low activity of this ubiquitination system is beneficial for patient survival. APEH (acylaminoacyl-peptide hydrolase) plays an important role in destroying oxidatively damaged proteins in living cells [36] and deletion of APEH locus have been found in small cell lung carcinoma [37] and renal cell carcinoma [38]. As a mammalian lectin, LGALS8 (galectin 8) can inhibit cell adhesion and induce apoptosis by binding to integrin a4αβ3 to modulate cell-matrix interactions [39], Therefore, high level of APEH and LGALS8 is beneficial to patient survival. CBR1 (carbonyl reductase 1) may reduce the effect of many anti-cancer drugs by metabolization, such as reducing the effect of doxorubicin in breast cancer patients [40] and attenuating the effect of arsenic trioxide in leukemia patients [41]. Thus, low level of CBR1 (CNV) is linked to better prognosis. In summary, this shared subnetwork implied that low activity of ubiquitin/proteasome degradation by low presence of UBA5/UFM1, and effective removal of oxidatively damaged proteins by APEH are linked to better prognosis.

### iref2/multinet1

RAB6A is a member of RAS oncogene family localized at Golgi apparatus [42], and RABGAP1 is a RAB6A activating protein playing a role in the coordination of microtubule and Golgi dynamics during the cell cycle [43]. CHML (CHM like, Rab escort protein 2) inhibits the geranylgeranylation reaction on RABs [44] and TMF1 (TATA element modulatory factor 1) is a conserved Golgi protein that binds to RAB6 and influences Golgi morphology [45], depletion of which blocks membrane transport among endosome, Golgi and ER [46]. Downregulation of TMF has been shown in solid tumors while overexpression of TMF significantly attenuated the growth of xenograft tumors [47]. Therefore, our prediction that low RAB oncogenic activity of by low CNV_RAB6A and mRNA_RABGAP1 and high level of their inhibitory factors CHML (mRNA, CNV) and CNV_TMF1 are beneficial for patient survival agrees well with previous studies.

Meanwhile, we would like to raise the point that one should carefully interpret and apply our results, because they are based on prognosis, which is from an outcome angle rather than from a causal angle. For example, we identified quite large amount of subnetworks that low ubiquitin/proteasome degradation is linked to better prognosis, which agrees well with current view of inhibiting ubiquitin/proteasome pathway proved to be effective strategies to treat various malignancies [48]. However, it does not necessarily mean that inhibiting ubiquitin/proteasome pathway will inhibit tumor growth, because there is still a possibility that low ubiquitin/proteasome activity is very likely an outcome of efficient ubiquitin/proteasome dependent mechanism to degrade oncogenic proteins (i.e. oncogenic products, oxidatively damaged proteins). It is also quite controversial about whether ER stress and UPR lead to cancer cell survival or death, which are context-dependent [49]. Thus, though our subnetworks biomarkers are quite efficient to predict TNBC patient outcome, one should carefully apply them when developing therapeutic strategies to specifically activate or inhibit members in the subnetworks.

Furthermore, we would like to advance the importance of integrating/combining multiple data types. Previous studies mainly focused on analyzing individual data type. Here, we found different data types from 66 and 376 genes significantly associated with TNBC and non-TNBC survival respectively. The major contribution of the 66 genes in TNBC came from DNA methylation (35 out of 66), while the contribution from both mRNA and CNV come from 16 genes. Such a difference may explain why combined molecular data type has a better prediction (highest C-index) than individual molecule data type (Figure 5). In contrast, 199 mRNA, 34 CNV, and 147 DNA methylation significantly associated with survival in non-TNBC, which also explained why mRNA was the best to predict survival for non-TNBC (highest C-index in Figure 5).

Additionally, it should be noted that our study still has some limitations that could guide future work. First, our predictive quality largely depends on the data itself and data quality we used. TCGA is the most comprehensive dataset available to us, but TNBC patients are quite limited and we had to leave miRNA information out because of insufficient samples left. Second, most of these TNBC patients are at early stage (stage I and II), and the lack of late stage TNBC patients (Stage III and IV) inevitably affects our final subnetworks. With more TNBC patients and the advancement of sequencing technology for various data types, our method could achieve better predictive results. Third, in statistical prediction, three other cross-validation methods (independent test, K-fold cross-validation, and jackknife test) are usually used to examine a predictor for its effectiveness in practical application and of the three test methods, the jackknife test is deemed the least arbitrary that can always yield a unique result for a given benchmark dataset [50]. Finally, as suggested by Chou [51], user-friendly and publicly accessible web-servers will significantly enhance the impacts in developing new prediction or analysis methods, thus we shall make efforts in our future work to provide a web-server for the identification method presented in here.

## MATERIALS AND METHODS

### Multi- dimensional genomic data

The multi-dimensional cancer-associated data sets containing clinical information, copy-number variation (CNV), promoter DNA methylation, mRNA expression were collected from TCGA Cancer Browser (https://genome-cancer.ucsc.edu/proj/site/hgHeatmap/). A brief summary of the data information is provided in Table 1. TN was determined as negative of clinical calls for estrogen receptor (EsR), progesterone (PgR) and human epidermal growth factor receptor 2 (HER2), while non-TN was determined by at least one positive of the above receptors. In the end, we collected 119 samples for TNBC and 583 samples for non-TNBC, respectively.

### Protein-protein interaction data

To date there is no single database of human protein-protein interactions with high sensitivity and specificity, yet we used three interaction networks with various types of interactions to allow for different false positive and false negative interactions described previously [22]. The data can be downloaded from:http://compbio-research.cs.brown.edu/pancancer/hotnet2/#!/data.

Basically, three networks were used: (1) HINT+HI2012, a combination of high-quality protein-protein interactomes from HINT [24] and HI-2012 [25] which consists of 40,783 interactions among 10,008 proteins; (2) iRefIndex [26], an integrated network from multiple data sources including various types of interactions (i.e. physical interaction, genetic inequality, (de)acetylation, (de)methylation, (de)phosphorylation, (de)ubiquitination) except for colocalizations and genetic interactions, which consists of 91,872 interactions among 12,338 proteins; and (3) MultiNet [27] a network that integrates multiple types of interactions, including protein-protein, phosphorylation, metabolic, signaling, genetic, and regulatory interactions from multiple databases, which consists of 109,597 interactions among 14,445 proteins.

### Data filtering

CNV profiling was filtered with 12099 expressed genes described previously [22], which include 12,081 genes with at least 3 RNA-Seq reads per sample in at least 70% of samples from syn1734155 plus 18 well-known cancer genes. Finally, CNV of a total of 11878 genes was consolidated in 119 TNBC and 583 non-TNBC samples. The DNA methylation profile was measured experimentally using either the Illumina Infimum Human DNA Methylation 450K (433 samples) or 27K (287 samples) platform. After merging the data from the two platforms, 19847 probes were mapped to 13474 genes in 123 TNBC samples and 595 non-TNBC samples. The mRNA expression profile was filtered with the 12099 expressed genes and 11,442 genes were left. For TNBC and non-TNBC, we defined the sample intersection across all platforms as the core sample set.

### Identification of novel survival-related subnetworks

Subnetwork signatures of survival-related genes were determined both by the scores of their genes, and the interactions among the genes. For each gene, we evaluated its effect on patient overall survival by taking into account the molecular features of mRNA expression, CNV, and promoter DNA methylation status. The R program “coxph” was used to fit a multivariate Cox proportional hazards model between each molecular feature and patient survival time adjusted for age, with the likelihood ratio test being used to estimate the significance. Only the features which passed the cutoff of *p* < 0.05 were considered to be survival-related. A heat score for each gene was calculated using the Equation (1) summarized as the sum negative natural logarithm of single survival-related molecular feature *p*-values, which is corresponded to Fisher's Method for combining *p*-values for (independent) statistical tests [23]. For DNA methylation, considering the fact that one gene may have multiple methylation loci, we only retained one CpG methylation probe that was most correlated with survival time.

$$\text{score}=-2\times \sum_{m}\log_{e}(p_{m}),m=\text{mRNA},\text{CNV},\text{methy}$$ (1)

The genes with a score > 0 were identified as survival-related genes. Subnetwork signatures in individual PPI network were discovered using HotNet2 [22, 23], which uses a heat diffusion process with the gene heat score as the input and a statistical test based algorithm (Figure 1A).

### Ranking subnetworks based on their prognostic power

For each subnetwork, we firstly assembled a multi-dimensional molecular profile by extracting three types of molecular features of its gene members from the core sample set. We then explored the predictive power of the subnetwork on patient overall survival using a Monte Carlo cross-validation and permutation testing procedure. Briefly, for the core sample set, we randomly split the samples into two groups: 80% as the training set and 20% as the test set. For the training set, we used the Cox proportional hazards model with ElasticNet [52], a modified L1 penalized log partial likelihood (LASSO) [53] for feature selection to train the models based on the molecular profile of individual subnetwork. ElasticNet was used for feature selection, because it combines penalty terms of LASSO and Ridge to compromise variable selection and group effect, considering the potential intrinsic relations among biologically relevant genes and widespread co-linearity of large-scale biological data [52]. The prognostic outcomes for the training set were used to determine the regression coefficients. These coefficients were then used to predict outcomes for patients in the test set and calculate the C-index. The above procedure was repeated 100 times to generate 100 C-indexes and the median C-index was used as the predictive value for each subnetwork. C-index has been frequently applied to evaluate risk prediction model and survival analysis: A C-index of 1 indicates perfect prediction accuracy, while a C-index of 0.5 is equivalent to a random guess. Our survival predictive models were evaluated based on a research framework which could be accessed in Synapse (doi:10.7303/syn1710282) (Figure 1B).

### Selection of important molecular features for prognostic model building and tumor stratification

When building the predictive model using the molecular features of each subnetwork, ElasticNet was used to select a small number of “important” features. Basically, 100 samplings of the training set could extract 100 important feature sets and the occurrence of each molecular feature was counted. Since the possibility of random selection bias for any given feature could be ruled out if the feature was consistently selected for, we only kept features occurring more than 5 times to construct our final predictive model for each subnetwork-based biomarker. And all the selected molecular features derived from the 39 subnetwork models were used for tumor stratification.

### Survival analysis

A risk score formula was established by weighting each of the selected features of the subnetwork by their estimated regression coefficients in the multivariable Cox regression analysis. With this risk score formula, we were able to calculate a subnetwork-based risk score (referred to as “snRS”) for each patient in the data set. Then the patients were classified into high-risk or low-risk groups using the median risk score as the cutoff. Survival differences between the low-risk and high-risk groups identified in each set were assessed by the Kaplan-Meier estimate and compared using the log-rank test. Univariate and multivariate analyses with Cox proportional hazards regression for overall survival were performed on the individual conventional clinical variables with and without the subnetwork-based signature in each dataset. Hazard ratios (HR) and 95% confidence intervals (CI) were calculated. All statistical tests were two-sided and performed with R software.

### Assessment of the prognostic power of diverse or combined molecular features-based models

First, a pre-selection step to keep the top significant features correlated with overall survival (univariate Cox model, likelihood ratio test, *p* < 0.05) was performed as Yuan's study [20]. Next, the ElasticNet [52]+Cox was applied to train the models in training set. Then the trained predictive models were used to predict outcomes for patients in the test set for the C-index calculation. For TNBC or non-TNBC core set, the above procedure was repeated 100 times from the 100 times of randomly splitting of the core set into training and test sets to generate 100 prognostic models and the corresponding C-indexes. From the 100 prognostic models, only the models with a C-index > 0.5 and a permutation test *p*-value < 0.05 based on a 100 survival-permutated data were retained for downstream analysis (22 models for TNBC and 31 models for non-TNBC). In total,28 mRNA, 43 CNV, and 48 DNA methylation features mapping to 66 genes were selected from the 22 TNBC models. In contrast, 199 mRNA, 34 CNV, and 147 DNA methylation mapping to 376 genes were found significantly associated with survival from the 31 non-TNBC models.

## SUPPLEMENTARY MATERIALS FIGURES AND TABLES








