# Supplementary material for: Identification of novel prognostic indicators for triple-negative breast cancer patients through integrative analysis of cancer genomics data and protein interactome data
Source: Oncotarget. 2016 Sep 27;7(44):71620–34. doi: 10.18632/oncotarget.12287 (PMC5342106; doi:10.18632/oncotarget.12287)
Supplement: Supplementary file 1 [file oncotarget-07-71620-s001.pdf]

Identification of novel prognostic indicators for triple-negative breast cancer patients through integrative analysis of cancer genomics data and protein interactome data

Supplementary Materials

S1 Text. Biological insights from network-based prognostic biomarkers

Among the subnetworks detected from the three different interaction networks, most strikingly, one subnetwork(inthint1/iref3/multinet2) was shared by the three PPI networks and two were shared by iRefIndex and MultiNet (iref2/multinet1, iref4/multinet6) (Figure 2). To further understand how these subnetwork biomarkers may be related to patient survival, we investigated individual gene using NCBI Entrez Gene [1] and literature search and performed functional enrichment analysis based on the known pathways or ontologies using Enrichr [2].

Such a thorough analysis identified several pathways known to be involved in TNBC such asiref4/multinet6, which contain BRCA1 interacting partners (RBBP7 by high mRNA, low methyl; BAP1 by high mRNA and CNV) together with transcriptional repressors (DHX30 by high CNV; HSPA2 by high mRNA, low methyl) and PIK3A signaling pathway (discuss in detail later). BRCA1 inactivation and PIK3CA high activity have been identified as key features in basal-like breast cancers (often referred to as TNBCs because of 75% overlap of mRNA profiles) [3]. Several metabolism-related subnetworks have also been identified, such as multinet3 (discuss in detail later) and multinet4/8/9/12. Members in multinet4/8/9/12 are mainly involved amino acid metabolism and enriched in mitochondria from Enrichr analysis. We found that elevated level of several synthetases involved in one carbon unit (MAT2B, MTHFD1, MTHFS, MTR), high presence of GPX1 (one of the most important antioxidant enzymes), and low presence of various enzymes involved in biosynthesis (CBS, PC, SPTLC2, NADSYN1, PAICS) were linked to better prognosis. Though we cannot reach a conclusion of which particular product/metabolic or activating or repressing which particular pathway is linked to better prognosis, such a phenomenon leads us to propose that these subnetworks together with various acetyl-CoA related enzymes in multient3 may be related to the Warburg effect in tumors, such as reactive oxygen (ROS) and biosynthesis [4]. Most importantly, we found quite large amount of a subnetworks related to GTPase, endoplasmic reticulum (ER)-Golgi-cell surface trafficking, ubiquitin/proteasome system, and complement system. Here, we discuss these top-ranked and shared subnetworks in detail (Figure 3).

iref12/inthint2/multinet11 and inthint3/multinet14

Complement related subnetworks stood out from our analysis. Complement system is well known to play key roles in innate and adaptive immunity [5]. Its role in cancer is a bit controversial: its has anti-tumor effect by direct complement attacked or complement-dependent cytotoxicity; it has tumor promoting effect by C5a induced ROS; tumors can also escape complement attack by expressing or recruiting complement regulators or inhibitors to inhibit the formation of terminal complement complex [6]. Previous studies have shown that that low activity of the classical complement pathway predicts short survival of patients with chronic lymphocytic leukaemia [7] and activation of complement and immune response pathways is associated with good prognosis in ER-negative breast cancer [8]. In fact, high presence of the majority of the genes in these subnetworks is linked to better survival (high mRNA_C1R, C1S, SERPING1, SERPINI1, CYTH1, VNN2; high CNV_ARF1, low methyl_CFB, CR1, CYTIP, ICAM3, ITGAM, ITGB2, PLAUR). In contrast, low presence of C1QA, C3, CFD, SURF2 by high methylation is also linked to better survival. Particularly, though high mRNA_C1R, C1S, SERPING1, SERPINI1 are linked to better survival, serpin family proteins (SERPING1, SERPINI1) apparently work as inhibitors of C1R and C1S [9]. Compared with inthint4/iref3/multinet12, which are more related to the peptidase activity, inthint3/multinet14 are more related to glycoprotein binding and guanyl-nucleotide exchange factor activity by Enrichr analysis. Therefore, our results may not fully support the idea that high complement activity is linked to better survival, but a sensitive and tightly regulated complement system is associated with patient survival.

inthint1/iref3/multinet2

We predicted that low UBA5 (mRNA, CNV), low UFM1 (CNV), high APEH (CNV), high LGALS8 (mRNA,CNV), and low CBR1 (CNV) were linked to better prognosis. UBA5 (ubiquitin like modifier activating enzyme 5) and UFM1 (ubiquitin-fold modifier 1) were identified as a novel protein-conjugating system, in which UBA5 worked as a novel E1-like enzyme to activate

C-terminal cleaved UFM1 by forming a high-energy thioester bond, and UFM1 was further transferred to E2-like enzyme UFC1 via thioester linkage [10]. Knockdown or depletion of UFL1 or UBA5 could lead to unfolded protein response (UPR), ER stress, and inhibited vesicle trafficking [11-13]. Knockdown of UBA5 has been shown to inhibit breast cancer cell growth [14], implying the low activity of this ubiquitination system is beneficial for patient survival. APEH (acylaminoacyl-peptide hydrolase) encodes the enzyme called acylpeptide hydrolase, which plays an important role in destroying oxidatively damaged proteins in living cells [15]. Deletion of APEH locus have been found in small cell lung carcinoma [16] and renal cell carcinoma [17]. Therefore, high activity of APEH is beneficial to patient survival by reducing the oxidative proteins. CBR1 (carbonyl reductase 1) has protective roles against xenobiotic carbonyls [18], thus it may reduce the effect of many anti-cancer drugs by metabolization, such as reducing the effect of doxorubicin in breast cancer patients [19, 20]. It could also attenuate the effect of arsenic trioxide in leukemia patients [21]. Thus, low level of CBR1 (CNV) is linked to better prognosis. As a mammalian lectin, LGALS8 (galectin 8) can inhibit cell adhesion and induce apoptosis by binding to integrin a4αβ3 to modulate cell-matrix interactions [22], which agrees with our prediction that high LGALS8 is linked to better survival. In summary, this shared subnetwork implied that low activity of ubiquitin/proteasome degradation by low presence of UBA5/UFM1, and effective removal of oxidatively damaged proteins by APEH are linked to better prognosis.

iref2/multinet1

RAB6A is a member of RAS oncogene family localized at Golgi apparatus [23], and RABGAP1 is a RAB6A activating protein playing a role in the coordination of microtubule and Golgi dynamics during the cell cycle [24]. CHML (CHM like, Rab escort protein 2) can facilitate the attachment of geranylgeranyl groups to several Rab proteins [25], while free CHML inhibits the prenylation reaction on RABs by RabGGTase by forming a CHML-RabGGTase complex [26]. TMF1 (TATA element modulatory factor 1) is a conserved Golgi protein binds to RAB6 and influences Golgi morphology [27], depletion of which blocks both retrograde membrane transport from endosome to trans-Golgi and RAB6-dependent cargo-specific transport from Golgi to ER (i.e. retention of glycosylation enzymes at Golgi) [28]. Downregulation of TMF has been shown in solid tumors while overexpression of TMF significantly attenuated the development and growth of xenograft tumors by decreasing proangiogenic genes in metabolic stress state (i.e. direct the ubiquitination and proteasomal degradation of p65/RelA) [29]. Therefore, our prediction that low RAB oncogenic activity of by low CNV_RAB6A and mRNA_RABGAP1 and high level of their inhibitory factors CHML (mRNA, CNV) and CNV_TMF1 are beneficial for patient survival agrees well with previous studies.

iref4/multinet6

This network contains a tightly regulated RAS signaling pathway. PIK3A is a well-known oncogene contributing to Warburg effect and affecting various pathways related to cancer (i.e. glucose transport and catabolism, cell adhesion, RAS signaling) and high CNV_PIK3A is commonly found in ovarian cancer biosynthesis [4, 30, 31]. Meanwhile, we reveal that low methyl_DGKZ and high mRNA_PPM1B are linked better survival. DKGZ (diacylglycerol kinase zeta) can inhibit both Ras-ERK and PKC pathways by regulating diacylglycerol (DAG) level [32, 33]. DKGZ can also bind to retinoblastoma protein (pRB) or act as a downstream effecter of pRB to reconstitute cell cycle arrest in pRB-null fibroblasts [34]. As a member of PP2C family of Ser/Thr protein phosphatases, PPM1B (phosphatase, Mg2+/Mn2+ dependent 1B) can dephosphorylate cyclin-dependent kinases (CDKs) to inhibit cell growth [35], IkappaB kinases (IKKs) to down-regulate cytokine-induced NF-kappaB activation [36], and stress-activated protein kinase (a member of MAPKs) to attenuate stress response [37]. Therefore, we revealed a subnetwork that high PIK3A by high mRNA and CNV is linked to worse survival, while high presence of DKGZ and PPM1B, two inhibitory proteins, are linked to better survival.

iref14/multinet3

Besides sharing three high-heat score genes, SRPRB, UQCR1, and USP19, iref14 is more transcription related while mMultinet3 is related to CoA metabolism. SPRRB (SRP receptor beta subunit) is a GTPase associated with ER membrane [38] and highly expressed in various tumors [39]. UQCRC1 (ubiquinol-cytochrome c reductase core protein I) is a critical component in mitochondrial respiratory electron transport chain [40]. It also plays a role in mitochondria-to-nucleus retrograde response invoked by depletion of mitochondrial DNA (mtDNA) and its expression is positively correlated with cytochrome c-oxidase encoded by mtDNA [41]. USP19 (ubiquitin specific peptidase 19) is an ER membrane-anchored deubiquitinating enzyme to rescue ER-associated degradation (ERAD) stubstrates from proteasomal degradation [42] and mount an appropriate response to hypoxia by rescuing hypoxia-inducible factor 1α (HIF-1α) from degradation [43]. Our prediction is low activity of SRPRB (mRNA, CNV) and high activity of UQCRC1 (CNV) and USP19 (mRNA, CNV) are linked to better survival, implying general low activities of GTPase (SRPRB) and ubiquitin/proteasome degradation (by high activity of deubiquitinating enzyme USP19) as well as normal mitochondrial respiratory electron transport chain (UQCRC1) are linked to better prognosis.

Furthermore, we would like to point out two one pathways highly associated with TNBC patient survival: iref56, semaphorins-plexins signaling pathway with RAS family small GTPases as downstream signaling [44] and iref18, nidogen proteins, members of extracellular matrix (ECM) which can interact with integrins and their expression is reduced in tumors[45]. Additionally, we would like to highlight some of the genes that we do not explain the relative subnetworks in detail but are recently reported as novel tumor suppressors linked to cancer prognosis, including PHLDA3 (a p53 target gene) by repressing AKT [46, 47], GGT7 by regulating anti-oxidative damage [48], and CTNNA1 (α-catenin) by inhibiting Wnt/β-catenin, NF-κB, Hippo-YAP, and Hedgehog signaling pathways [49].

REFERENCES

1. Maglott D, Ostell J, Pruitt KD, Tatusova T. Entrez Gene: gene-centered information at NCBI. Nucleic Acids Res. 2011; 39:D52–D57.
2. Chen EY, Tan CM, Kou Y, Duan Q, Wang Z, Meirelles GV, Clark NR, Ma›ayan A. Enrichr: interactive and collaborative HTML5 gene list enrichment analysis tool. BMC Bioinformatics. 2013; 14:128.
3. Cancer Genome Atlas N. Comprehensive molecular portraits of human breast tumours. Nature. 2012; 490:61–70.
4. Heiden MGV, Cantley LC, Thompson CB. Understanding the Warburg Effect: The Metabolic Requirements of Cell Proliferation. Science. 2009; 324:1029–1033.
5. Carroll MC. The complement system in regulation of adaptive immunity. Nat Immunol. 2004; 5:981–986.
6. Ricklin D, Hajishengallis G, Yang K, Lambris JD. Complement: a key system for immune surveillance and homeostasis. Nat Immunol. 2010; 11:785–797.
7. Varga L, Czink E, Miszlai Z, Paloczi K, Banyai A, Szegedi G, Fust G. Low activity of the classical complement pathway predicts short survival of patients with chronic lymphocytic leukaemia. Clin Exp Immunol. 1995; 99:112–116.
8. Teschendorff AE, Miremadi A, Pinder SE, Ellis IO, Caldas C. An immune response gene expression module identifies a good prognosis subtype in estrogen receptor negative breast cancer. Genome Biol. 2007; 8.
9. Gaboriaud C, Thielens NM, Gregory LA, Rossi V, Fontecilla-Camps JC, Arlaud GJ. Structure and activation of the C1 complex of complement: unraveling the puzzle. Trends Immunol. 2004; 25:368–373.
10. Komatsu M, Chiba T, Tatsumi K, Iemura SI, Kominami E, Natsume T, Tanaka K. A novel protein-conjugating system for Ufm1, a ubiquitin-fold modifier. Cell Struct Funct. 2004; 29:97–97.
11. Zhang YH, Zhang MS, Wu JC, Lei GH, Li HL. Transcriptional Regulation of the Ufm1 Conjugation System in Response to Disturbance of the Endoplasmic Reticulum Homeostasis and Inhibition of Vesicle Trafficking. Plos One. 2012; 7.
12. Zhang M, Zhu X, Zhang Y, Cai Y, Chen J, Sivaprakasam S, Gurav A, Pi W, Makala L, Wu J, Pace B, Tuan-Lo D, Ganapathy V, Singh N, Li H. RCAD/Ufl1, a Ufm1 E3 ligase, is essential for hematopoietic stem cell function and murine hematopoiesis. Cell Death Differ. 2015; 22: 1922–1934.
13. Cai YF, Pi WH, Sivaprakasam S, Zhu XB, Zhang MS, Chen JJ, Makala L, Lu CW, Wu JC, Teng Y, Pace B, Tuan D, Singh N, Li HL. UFBP1, a Key Component of the Ufm1 Conjugation System, Is Essential for Ufmylation-Mediated Regulation of Erythroid Development. Plos Genet. 2015; 11.
14. Yoo HM, Kang SH, Kim JY, Lee JE, Seong MW, Lee SW, Ka SH, Sou YS, Komatsu M, Tanaka K, Lee ST, Noh DY, Baek SH, Jeon YJ, Chung CH. Modification of ASC1 by UFM1 Is Crucial for ER alpha Transactivation and Breast Cancer Development. Mol Cell. 2014; 56:261–274.
15. Fujino T, Watanabe K, Beppu M, Kikugawa K, Yasuda H. Identification of oxidized protein hydrolase of human erythrocytes as acylpeptide hydrolase. Bba-Protein Struct M. 2000; 1478:102–112.
16. Naylor SL, Marshall A, Hensel C, Martinez PF, Holley B, Sakaguchi AY. The Dnf15s2 Locus at 3p21 Is Transcribed in Normal Lung and Small Cell Lung-Cancer. Genomics. 1989; 4:355–361.
17. Erlandsson R, Bergerheim USR, Boldog F, Marcsek Z, Kunimi K, Lin BYT, Ingvarsson S, Castresana JS, Lee WH, Lee E, Klein G, Sumegi J. A Gene near the D3f15s2 Site on 3p Is Expressed in Normal Human Kidney but Not or Only at a Severely Reduced Level in 11 of 15 Primary Renal-Cell Carcinomas (Rcc). Oncogene. 1990; 5:1207–1211.
18. Oppermann U. Carbonyl reductases: The complex relationships of mammalian carbonyland quinone-reducing enzymes and their role in physiology. Annu Rev Pharmacol. 2007; 47:293–322.
19. Lal S, Sandanaraj E, Wong ZW, Ang PCS, Wong NS, Lee EJD, Chowbay B. CBR1 and CBR3 pharmacogenetics and their influence on doxorubicin disposition in Asian breast cancer patients. Cancer Sci. 2008; 99:2045–2054.
20. Mitani T, Ito Y, Harada N, Nakano Y, Inui H, Ashida H, Yamaji R. Resveratrol Reduces the Hypoxia-Induced Resistance to Doxorubicin in Breast Cancer Cells. J Nutr Sci Vitaminol. 2014; 60:122–128.
21. Jang M, Kim Y, Won H, Lim S, Jyothi KR, Dashdorj A, Min YH, Kim SY, Shokat KM, Ha J, Kim SS. Carbonyl Reductase 1 Offers a Novel Therapeutic Target to Enhance Leukemia Treatment by Arsenic Trioxide. Cancer Res. 2012; 72:4214–4224.
22. Hadari YR, Arbel-Goren R, Levy Y, Amsterdam A, Alon R, Zakut R, Zick Y. Galectin-8 binding to integrins inhibits cell adhesion and induces apoptosis. J Cell Sci. 2000; 113: 2385–2397.
23. Opdam FJM, Echard A, Croes HJE, van den Hurk JAJM, van de Vorstenbosch RA, Ginsel LA, Goud B, Fransen JAM. The small GTPase Rab6B, a novel Rab6 subfamily member, is cell-type specifically expressed and localised to the Golgi apparatus. J Cell Sci. 2000; 113:2725–2735.

24. Cuif MH, Possmayer F, Zander H, Bordes N, Jollivet F, Couedel-Courteille A, Janoueix-Lerosey I, Langsley G, Bornens M, Goud B. Characterization of GAPCenA, a GTPase activating protein for Rab6, part of which associates with the centrosome. Embo J. 1999; 18: 1772–1782.
25. Cremers FP, Armstrong SA, Seabra MC, Brown MS, Goldstein JL. REP-2, a Rab escort protein encoded by the choroideremia-like gene. J Biol Chem. 1994; 269:2111–2117.
26. Anant JS, Desnoyers L, Machius M, Demeler B, Hansen JC, Westover KD, Deisenhofer J, Seabra MC. Mechanism of Rab geranylgeranylation: formation of the catalytic ternary complex. Biochemistry. 1998; 37:12559–12568.
27. Fridmann-Sirkis Y, Siniossoglou S, Pelham HR. TMF is a golgin that binds Rab6 and influences Golgi morphology. BMC Cell Biol. 2004; 5:18.
28. Yamane J, Kubo A, Nakayama K, Yuba-Kubo A, Katsuno T, Tsukita S, Tsukita S. Functional involvement of TMF/ARA160 in Rab6-dependent retrograde membrane traffic. Exp Cell Res. 2007; 313:3472–3485.
29. Abrham G, Volpe M, Shpungin S, Nir U. TMF/ARA160 downregulates proangiogenic genes and attenuates the progression of PC3 xenografts. Int J Cancer. 2009; 125:43–53.
30. Shayesteh L, Lu YL, Kuo WL, Baldocchi R, Godfrey T, Collins C, Pinkel D, Powell B, Mills GB, Gray JW. PIK3CA is implicated as an oncogene in ovarian cancer. Nat Genet. 1999; 21:99–102.
31. Thorpe LM, Yuzugullu H, Zhao JJ. PI3K in cancer: divergent roles of isoforms, modes of activation and therapeutic targeting. Nat Rev Cancer. 2015; 15:7–24.
32. Merida I, Andrada E, Gharbi SI, Avila-Flores A. Redundant and specialized roles for diacylglycerol kinases alpha and zeta in the control of T cell functions. Sci Signal. 2015; 8.
33. Topham MK, Prescott SM. Diacylglycerol kinase zeta regulates Ras activation by a novel mechanism. J Cell Biol. 2001; 152:1135–1143.
34. Los AP, Vinke FP, de Widt J, Topham MK, van Blitterswijk WJ, Divecha N. The retinoblastoma family proteins bind to and activate diacylglycerol kinase zeta. J Biol Chem. 2006; 281:858–866.
35. Cheng A, Ross KE, Kaldis P, Solomon MJ. Dephosphorylation of cyclin-dependent kinases by type 2C protein phosphatases. Genes Dev. 1999; 13:2946–2957.
36. Prajapati S, Verma U, Yamamoto Y, Kwak YT, Gaynor RB. Protein phosphatase 2Cbeta association with the IkappaB kinase complex is involved in regulating NF-kappaB activity. J Biol Chem. 2004; 279:1739–1746.
37. Nguyen AN, Shiozaki K. Heat shock-induced activation of stress MAP kinase is regulated by threonine- and tyrosine-specific phosphatases. Gene Dev. 1999; 13:1653–1663.
38. Miller JD, Tajima S, Lauffer L, Walter P. The Beta-Subunit of the Signal Recognition Particle Receptor Is a Transmembrane Gtpase That Anchors the Alpha-Subunit, a Peripheral Membrane Gtpase, to the Endoplasmic-Reticulum Membrane. J Cell Biol. 1995; 128:273–282.
39. Zhang YQ, Li QL, Zhu F, Cui JH, Li KN, Li Q, Wang RA, Wang WY, Wang WH, Yan W. Subcellular localization of APMCF1 and its biological significance of expression pattern in normal and malignant human tissues. J Exp Clin Canc Res. 2009; 28.
40. Hoffman GG, Lee S, Christiano AM, Chunghonet LC, Cheng W, Katchman S, Uitto J, Greenspan DS. Complete Coding Sequence, Intron-Exon Organization, and Chromosomal Location of the Gene for the Core-I Protein of Human Ubiquinol-Cytochrome-C Reductase. Journal of Biological Chemistry. 1993; 268:21113–21119.
41. Kulawiec M, Arnouk H, Desouki MM, Kazim L, Still I, Singh KK. Proteomic analysis of mitochondria-to-nucleus retrograde response in human cancer cells. Cancer Biol Ther. 2006; 5:967–975.
42. Hassink GC, Zhao B, Sompallae R, Altun M, Gastaldello S, Zinin NV, Masucci MG, Lindsten K. The ER-resident ubiquitin-specific protease 19 participates in the UPR and rescues ERAD substrates. Embo Rep. 2009; 10:755–761.
43. Altun M, Zhao B, Velasco K, Liu H, Hassink G, Paschke J, Pereira T, Lindsten K. Ubiquitin-specific protease 19 (USP19) regulates hypoxia-inducible factor 1alpha (HIF-1alpha) during hypoxia. J Biol Chem. 2012; 287:1962–1969.
44. Worzfeld T, Offermanns S. Semaphorins and plexins as therapeutic targets. Nat Rev Drug Discov. 2014; 13: 603–621.
45. Ulazzi L, Sabbioni S, Miotto E, Veronese A, Angusti A, Gafa R, Manfredini S, Farinati F, Sasaki T, Lanza G, Negrini M. Nidogen 1 and 2 gene promoters are aberrantly methylated in human gastrointestinal cancer. Mol Cancer. 2007; 6.
46. Kawase T, Ohki R, Shibata T, Tsutsumi S, Kamimura N, Inazawa J, Ohta T, Ichikawa H, Aburatani H, Tashiro F, Taya Y. PH Domain-Only Protein PHLDA3 Is a p53-Regulated Repressor of Akt. Cell. 2009; 136:535–550.
47. Ohki R, Saito K, Chen Y, Kawase T, Hiraoka N, SaigawaR, Minegishi M, Aita Y, Yanai G, Shimizu H, Yachida S, Sakata N, Doi R, Kosuge T, Shimada K, Tycko B, et al. PHLDA3 is a novel tumor suppressor of pancreatic neuroendocrine tumors. P Natl Acad Sci USA. 2014; 111:E2404-E2413.
48. Bui TT, Nitta RT, Kahn SA, Razavi SM, Agarwal M, Aujla P, Gholamin S, Recht L, Li G. gamma-Glutamyl transferase 7 is a novel regulator of glioblastoma growth. Bmc Cancer. 2015; 15.
49. Sun Y, Zhang J, Ma L. alpha-catenin. A tumor suppressor beyond adherens junctions. Cell Cycle. 2014; 13:2334–2339.

Supplementary Table S1: Detail information for the 5 subnetworks detected in HINT + HI2012network. See Supplementary_Table_S1

Supplementary Table S2: Detail information for the 20 subnetworks detected in iRefIndex network. See Supplementary_Table_S2

Supplementary Table S3: Detail information for the 15 subnetworks detected in MultiNetnetwork. See Supplementary_Table_S3
